# Tensile and Compression Strength Prediction and Validation in 3D-Printed Short-Fiber-Reinforced Polymers

**DOI:** 10.3390/polym15173605

**Published:** 2023-08-30

**Authors:** Timothy Russell, David A. Jack

**Affiliations:** Department of Mechanical Engineering, Baylor University, Waco, TX 76798, USA; timothy_russell@alumni.baylor.edu

**Keywords:** Jeffery model, closure, fiber orientation, short-fiber composites, structural properties, large area additive manufacturing

## Abstract

In the current study, a methodology is validated for predicting the internal spatially varying strength properties in a single 3D-printed bead composed of 13%, by weight, carbon-fiber-filled acrylonitrile butadiene styrene. The presented method allows for the characterization of the spatially varying microstructural behavior yielding a local anisotropic stiffness and strength that can be integrated in a finite element framework for a bulk estimate of the effective stiffness and strength. The modeling framework is presented with a focus on composite structures made from large area additive manufacturing (LAAM). LAAM is an extrusion-based process yielding components on the order of meters, with a typical raster size of 10 mm. The presented modeling methods are applicable to other short-fiber-reinforced polymer processing methods as well. The results provided indicate the modeling framework yields results for the effective strength and stiffness that align with experimental characterization to within ∼1% and ∼10% for the longitudinal compressive and tensile strength, respectively, and to within ∼3% and ∼50% for the longitudinal compressive and tensile stiffness, respectively.

## 1. Introduction

The development of better manufacturing techniques promotes the development of new engineering materials, and vice versa. This concept is well embodied in the area of 3D-printed short-fiber-reinforced polymers (SFRPs). These materials offer the attractive combination of good mechanical properties compared to virgin polymers and a relatively easy processing. In addition, they help enable large area additive manufacturing (LAAM), which often uses carbon fiber to provide crucial thermal and mechanical property advantages (see, e.g., [1]). However, 3D-printed SFRPs introduce added difficulty in engineering design since their anisotropic properties are more complicated to model. Additive manufacturing allows one to customize tool paths to tailor that anisotropy to one’s advantage (for example, one can orient the way a part is built so that it has greater stiffness and strength in a load-bearing direction), but good methods for being able to predict these anisotropic properties must be established so that one can generate designs with high confidence.

The anisotropic properties of 3D-printed SFRPs are a function of the internal, spatially varying fiber orientation state. Thus, predicting the properties of SFRP parts oftentimes involves the problem of predicting their internal fiber orientation state first, then predicting the properties as functions of that orientation state. Research on predicting the spatially varying fiber orientation state in a fluid flow has its roots going back about a hundred years ago to George B. Jeffery [2]. The works of Charles Tucker III and his students in the past few decades have contributed greatly to the current understanding of this topic as well. Tucker and his student Suresh Advani brought orientation tensors to light as a general but very compact way to describe orientation states, and these have since become mainstream, allowing for the prediction of material properties as functions of those orientation tensors [3]. Folgar and Tucker [4] introduced a fiber interaction parameter to allow for the modeling of dense suspensions, and in the past decade, multiple models have been developed to advance the initial work of Folgar and Tucker to implement fiber orientation kinematics for industrial flow processes (see, e.g., [5,6,7]), each of which uses the orientation tensor approach to characterize the fiber alignment state. This topic is discussed in detail in Section 4 while introducing the mathematical framework. Modern modeling techniques now give the ability to predict the final properties of SFRP parts based on the processing conditions used to build said parts. We wish to bring this capability to light in the additive manufacturing industry so that SFRP parts can be evaluated prior to printing, saving time and money and improving engineers’ confidence level in their designs.

The next two sections of this paper discuss experimental specimen preparation and testing, respectively. A miniature LAAM system was constructed for printing specimens. Section 4 then covers some of the mathematical models used to make predictions. Finally, Section 5 compares the experimental findings with the predicted structural behavior. The novelty of the present work is the experimental validation of a methodology, also demonstrated in [8], which fully integrates fiber orientation kinetics with structural property predictions for additively manufactured composites made from the LAAM process. This work is extended to compare against experimental observations and highlights the need to properly account for both the proper fiber orientation kinetics slowness parameter and the void content within the deposited material, the latter of which is able to be neglected in other industrial processes that study fiber orientation.

## 2. Specimen Preparation

### 2.1. Baylor’s Large Area Additive Manufacturing System

For fabricating test specimens, a miniature LAAM system was constructed at Baylor University. This system has been utilized in a variety of studies by various researchers (see e.g., [9,10]). The LAAM system has a print area of approximately 1.2 m by 1.2 m and the extruder has approximately 15 cm of vertical travel space. The bed can also be manually unfastened from the perforated tubing it is attached to, lowered, and refastened for added vertical space. The materials for the main structure of the LAAM system include cold-rolled steel railings, a 6.4 mm aluminum plate for the print bed, and 76 mm steel tubing for the frame. The LAAM system also includes a heated print bed composed of multiple zones.

**Figure 1:**
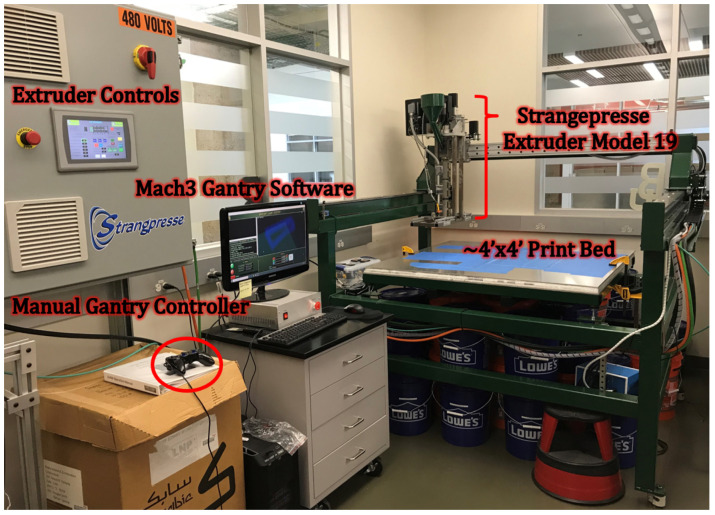
Baylor’s large area additive manufacturing (LAAM) system.

**Figure 2:**
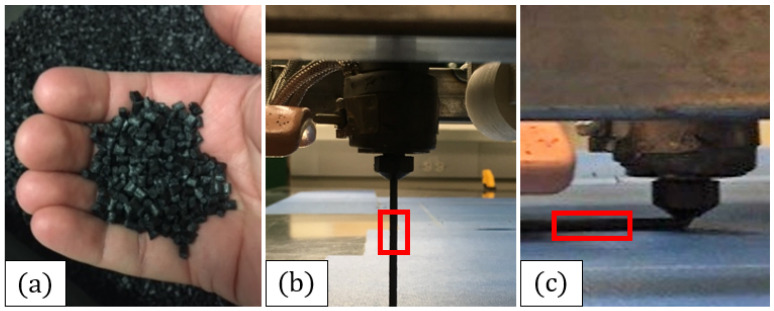
Three states of printed material: (**a**) a preprocessed pellet, (**b**) extrudate, and (**c**) a deposited bead.

The extruder on the Baylor LAAM system is a Strangpresse Extruder Model 19, which has a pellet-fed hopper at the top which was a custom-made part fabricated at Baylor University. The Strangpresse extruder shown Figure 1 is about 1 m tall with the hopper. The Strangpresse human–machine interface (HMI) allows an operator to adjust the temperatures in three zones along the length of the extruder as well as the extruder screw revolutions per minute (RPM) to control the deposition rate. The gantry system used for the motion control of the extruder is a Magnum II system from Precision Plasma LLC. The Magnum II system was made from an assembly kit for computer numerical control (CNC) plasma cutters, but its ability to move in three dimensions makes it suitable for custom LAAM systems as well, if the plasma cutter head is replaced with a suitable extruder. The gantry system is controlled using the Mach software Mach3 by Newfangled Solutions (Livermore Falls, ME, USA) managed on a local computer. All the experimental specimens for this study were fabricated using G-codes manually written by the author and ported to Mach3. The individual pellets, shown in Figure 2a, are fed into the hopper and then the filament is extruded, as shown in Figure 2b, and then deposited on to the print bed as shown in Figure 2c. During each of these various steps, the polymer is subjected to shearing and elongation, thus increasing the potential for fiber damage. The beads in the present study were composed of 13%, by weight, carbon-fiber-reinforced acrylonitrile butadiene styrene (CF-ABS).

### 2.2. Reduction of Additively Manufactured Bead to a Test Specimen

For the fiber length distribution and aspect ratio characterization, single beads of acrylonitrile butadiene styrene (ABS) pellets with 13% carbon fiber, by weight, were printed at 250 ${}^{{}^{\circ}}$C on a 95 ${}^{\circ }$C bed. Single beads were selected so as to keep the present focus on the individual deposited bead. Future studies proposed by the authors will focus on multiple deposited beads to study issues presented due to the presence of an interface. Specimens were gathered at the three different points in the printing process indicated in Figure 2, the preprocessed pellet, predeposition extrudate, and the deposited bead. Obtaining the fiber aspect ratio at each stage enables one to identify the most damaging part of the printing process and quantify its effect on the material property predictions. It was found that the volume fraction of fibers was consistent across all three stages of the process and over multiple samples processed over various batches. Thus, it was reasonably assumed that the fibers were well dispersed in the end product, but this did not suggest that there would not exist a small number of local variations in the fiber packing density as demonstrated in the companion study [11]. The present paper is focused on local variations in orientation and for simplicity, assumes that the volume fraction of fibers is homogeneous.

For the strength specimens, the 13% CF-ABS was dried for 4 h at 82 ${}^{\circ }$C prior to printing and stored in an environmental chamber with a −40 ${}^{\circ }$C dew point. For printing the specimens, the three extruder temperature zones were set to 200 ${}^{\circ }$C, 205 ${}^{\circ }$C, and 210 ${}^{\circ }$C, and the extruder RPM was set to 2250 with a nozzle-to-bed distance of 3 mm.

**Figure 3:**
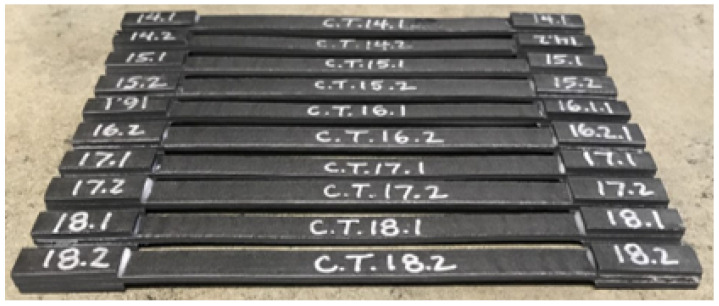
Photo of 13% CF-ABS tensile specimens prior to testing.

After printing, the upper surface of the beads were milled to remove the roughness of the surface of the bead. After machining, the specimens were sectioned with a Buehler IsoMet${}^{\mathrm{TM}}$ Low Speed Precision Cutter (Lake Bluff, IL, USA). The tensile specimens were 165 mm in length with 25 mm long tabs and a nominal thickness of 3 mm. The dimensions of the compression specimens were 81 mm in length with 38 mm long tabs and a nominal thickness of 3 mm. Specimen dimensions were of a single deposited raster and the length dimension was along the raster deposition dimension. After the specimens were cut, they were then cleaned, and the tabs were adhered using a Hysol 3039A adhesive and cured following the manufacturer’s recommended curing cycle. Tensile specimens were then heat-treated at 110 ${}^{\circ }$C for 10–15 min to reduce the warpage induced by milling the top surface and adhesive bonding. For the compression samples there was no measurable warpage, so they were not subjected to any heat treatment. After assembly of the tabs, samples were milled on their sides to ensure a constant, rectangular cross section and dimensional consistency between specimens. Figure 3 shows the prepared tensile specimens.

## 3. Experimental Characterization of Fiber-Reinforced Additive Manufactured Composite

The key experimental results of this paper are discussed in this section. Each subsection both describes an experimental setup and presents the results from that experimental setup. The fiber length distribution and aspect ratio study is discussed first. Next, the tensile stiffness and strength results are given. Finally, the compression stiffness and strength results are presented.

### 3.1. Fiber Length Distribution and Aspect Ratio Characterization

The process of characterizing the fiber length distribution consisted of five major steps. The first involved obtaining specimens. Specimens were gathered from the three points in the printing process shown in Figure 2. That is, a pellet, a 25.4 mm extrudate specimen, and a 25.4 mm printed specimen were gathered, with the printed specimen being of primary importance in this study. The second step was to isolate the fibers from each sample without damaging them. The third step was to capture micrographs of the fibers, stitch them together, and measure the fibers using a custom MATLAB code. The fourth and final step was to plot the fiber length distribution, get the number-average and weight-average fiber aspect ratios, and predict other material properties of interest.

**Figure 4:**
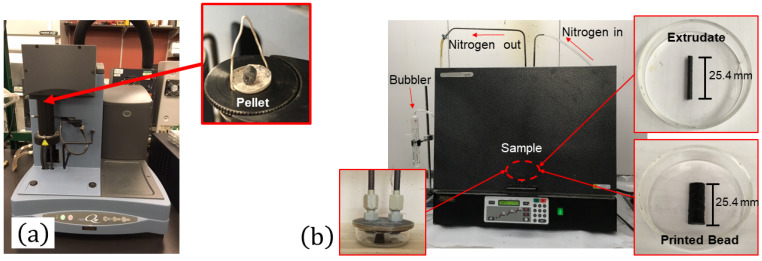
Burn-off testing setups. (**a**) TA Instruments Q50 TGA machine with a 13% CF-ABS pellet. (**b**) Ney Vulcan 3-1750 box furnace and 1-inch extrudate and printed specimens. These specimens were enclosed in a Petri dish with a custom-machined aluminum lid and inserted into the furnace.

Extracting the individual fibers from the matrix was accomplished by using a thermal digestion of each specimen. The complete test setup is shown in Figure 4, and the test setup where pellets were placed in a TA Instruments Q50 Thermogravimetric Analyzer (TGA) is shown in Figure 4a (New Castle, DE, USA). The burn-off procedure consisted of (1) ramping up the temperature to 600 ${}^{\circ }$C, (2) holding it isothermal for 1 h in nitrogen at 600 ${}^{\circ }$C, and (3) holding it isothermal for 10 min in air at 600 ${}^{\circ }$C. The inert, nitrogen atmosphere prevented the carbon fibers from degrading. However, to get rid of remaining residue after step 2, step 3 was added so that the fibers could completely separate from each other. Adding step 3 is not ideal because the fibers may degrade slightly, skewing the measured fiber length distribution. Therefore, a correction factor was applied to the measurement data to mitigate these effects, as discussed in [12].

To decrease the effect of cut fibers on skewing the fiber length distribution of the extrudate and printed specimens, 25.4 mm specimens were used since they were relatively large compared to the nominal length of a fiber, 100∼500 μm. These specimens were too large to fit in the TGA machine though, so a Ney Vulcan 3-1750 box furnace (Scotia, NY, USA) was used as shown in Figure 4b. The specimens were enclosed in a Petri dish with an aluminum lid. Two pipes attached to the aluminum lid purged the enclosure with nitrogen. A similar burn-off procedure as for the pellet was repeated for the extrudate and printed specimens.

**Figure 5:**
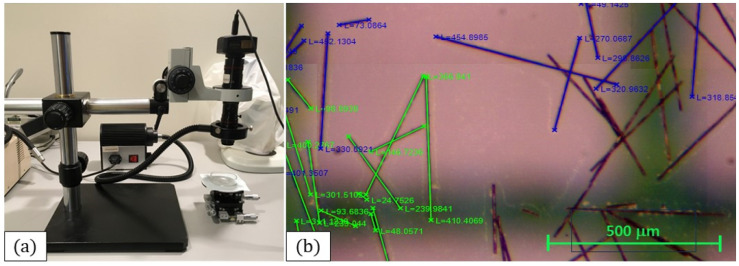
(**a**) An MZ7 microscope, used to capture micrographs of the fibers in a Petri dish. (**b**) Fibers being measured in a stitched micrograph. Green graph paper was placed under the Petri dish to provide a helpful frame of reference. The measurements in blue were measured in a current session, whereas the green measurements were from a previous measurement session.

The third step in characterizing the fiber length distribution was to capture images of the fibers under an MZ7 microscope, (Scienscope, New Haven, CT, USA) shown in Figure 5a, and stitch them together. After burn-off testing, the burn-off specimens were dropped into beakers of acetone and water and dispersed using a Branson Digital Sonifier 450 (Branson, MO, USA). The solutions were poured into Petri dishes before the dispersed fibers had time to settle completely. After pouring the dispersed fibrous solution into a Petri dish, the Petri dish was examined under a microscope and several micrographs were captured and stitched together. Figure 5b shows a micrograph, stitched in Adobe Photoshop CC 2017 and the resulting image was used for the fiber length measuring.

After stitching, a custom code written in the MATLAB environment was used to measure the fibers. The code allows one to measure a fiber by performing a mouse-click on both ends of the fiber. First, however, a microscopic ruler must be measured to find the proper pixel-to-millimeter scaling factor. Once a fiber is measured, the length of the fiber is automatically scaled and logged. The code allows a user to measure several fibers at once, close out of the measurement session, and return to it at a later time without losing any data.

**Figure 6:**
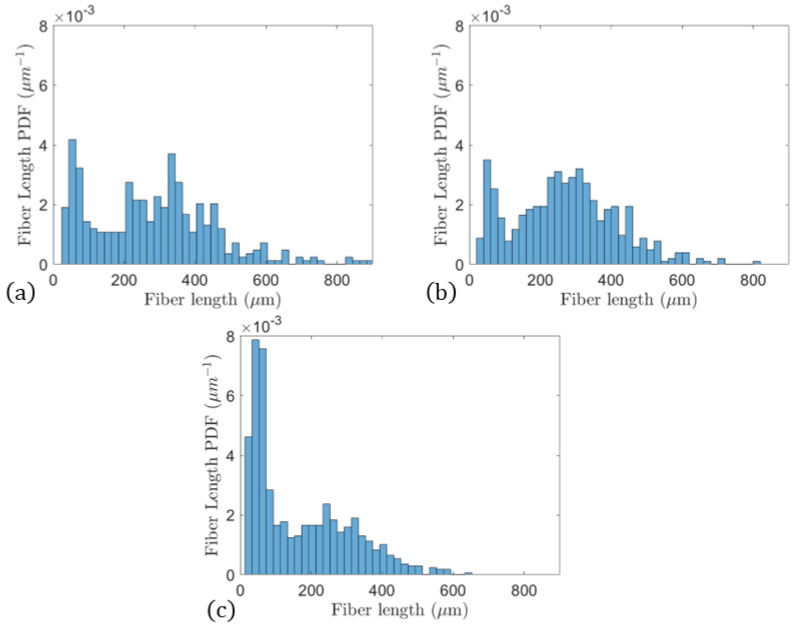
Fiber length distributions from (**a**) the preprocessed pellet, (**b**) the extrudate (predeposition), and (**c**) the printed bead (postdeposition).

The fourth step was to plot the probability distribution function (PDF) of the fiber length and calculate other quantities of interest. Figure 6 shows the PDF of the fiber length, with the PDF being cast in units of μm${}^{-1}$ for the three manufacturing stages. From the data in Figure 6, the number- and weight-average fiber lengths were calculated, respectively, by

(1) $$\begin{array}{ccc} L_{n} & = & \frac{\sum N_{i}L_{i}}{\sum N_{i}} \end{array}$$

(2) $$\begin{array}{ccc} L_{w} & = & \frac{\sum N_{i}L_{i}^{2}}{\sum N_{i}L_{i}} \end{array}$$

In the above equations, $N_{i}$ is the number of fibers in the *i*th histogram bin, and $L_{i}$ is the average fiber length within the *i*th histogram bin. Similarly, the number- and weight-average aspect ratios were approximated, respectively, as

(3) $$\begin{array}{ccc} a_{rn} & = & \frac{L_{n}}{d_{n}} \end{array}$$

(4) $$\begin{array}{ccc} a_{rw} & = & \frac{L_{w}}{d_{w}} \end{array}$$

where $d_{n}$ and $d_{w}$ are taken from [12]. The results for the fiber diameters, lengths, and aspect ratios for both the weight and number averages are given in Table 1 for this study. Note, these data were corrected for fiber degradation due to thermal heating in the presence of oxygen to remove the polymer matrix as detailed in [12].

### 3.2. Stiffness and Strength Characterization

This section discusses the stiffness and strength testing. The results for the tensile properties are given first, followed by the compression results.

#### 3.2.1. Tensile Testing

**Figure 7:**
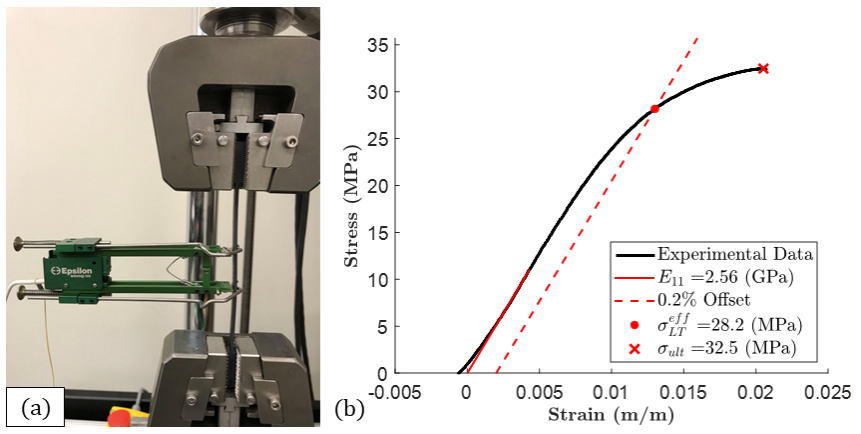
(**a**) Tensile testing setup with a 1 inch Epsilon 3542-0100-100-HT2 extensometer. (**b**) Tensile stress–strain plot for 13% CF-ABS.

A Test Resources 810 Series fatigue tester with a F2500-B load cell was used for the tensile testing. Figure 7a shows a mounted specimen with an extensometer for measuring strain. The specimens were tested at a speed of 0.059 in/min with a data collection rate of 20 Hz and at a temperature of approximately 21 ${}^{\circ }$C (70 ${}^{\circ }$F). Three width and three thickness measurements were taken for each of the five specimens used in the tensile study using micrometers in or near the gage region prior to testing. The width and thickness measurements were averaged to obtain the cross-sectional area of each specimen, from which the stress was calculated. A typical tensile stress–strain plot is shown in Figure 7b.

The effective longitudinal tensile stiffness of the tensile specimens was defined as $E_{LT}^{eff}$, where the *LT* subscript indicates the longitudinal tensile component and the *eff* superscript indicates the bulk stiffness of the locally varying stiffness. The effective longitudinal tensile stiffness was defined over the strain range 0.003–0.005. In addition, the effective longitudinal yield strength, defined as the term $\sigma_{LT}^{eff}$, was determined by using an offset as defined in ASTM D638 [13]. In the present study, a 0.2% offset was selected due to the initial nonlinear loading region and to align with the literature for which the models were based upon. That is, a line parallel to the elastic region was extended from (0, 0.002) up to the stress–strain curve, and the stress at the intersection was taken as $\sigma_{LT}^{eff}$. A toe region sometimes appeared at the beginning of the stress–strain curves, which can be seen in Figure 7b. Therefore, the data were horizontally shifted to where the $E_{LT}^{eff}$ lines would intersect the origin, prior to applying the 0.2% offset method. A summary of the results of four tests is given in Table 2, where $\bar{x}$, $s_{n-1}$, and $C_{V}$ are, respectively, the average, standard deviation, and coefficient of variation for the five specimens studied.

#### 3.2.2. Confined Compression Testing

**Figure 8:**
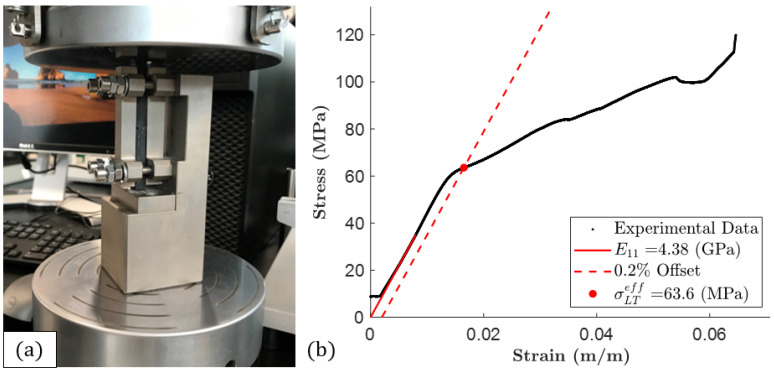
(**a**) A compression specimen mounted in a modified ASTM D695 compression test fixture (a Boeing BSS 7260) and between two platens. (**b**) A typical compression stress–strain plot for a 13% CF-ABS specimen.

The same Test Resources 810 Series fatigue tester with the same F2500-B load cell was also used for compression testing. Figure 8a shows a specimen in a modified ASTM D695 compression test fixture (Boeing BSS 7260) [14], which was placed between two compression platens mounted on the testing machine. Three width measurements of the five specimens were taken for each compression specimen, two on either end and one in the middle. The compression specimens had tabs covering most of their length, so only one thickness measurement was taken in the middle of the specimens, and this was done with calipers since the micrometer anvils did not fit between the tabs. The average width and thickness measurements were used to calculate the specimen’s cross-sectional areas from which the stress was derived. The length of each of the five specimens was also measured and used to calculate the approximate strain. Before initiating a test, the upper compression platen was lowered in contact with the specimen. Each test was conducted at 0.051 in/min (1.3 mm/min, as called for by ASTM D695) with a data collection rate of 20 Hz and an environmental temperature of about 21–22 ${}^{\circ }$C (70–72 ${}^{\circ }$F).

A typical compression stress–strain curve is shown in Figure 8b. Five compression tests were performed. The toe region of each curve was trimmed, this time by determining the stiffness over the stress range 25–35 MPa and horizontally shifting the data. In addition, the effective longitudinal compression yield strength, $\sigma_{LC}^{eff}$, was determined using a 0.2% strain offset. The results of the compression tests are given in Table 3. The ultimate compression strength is not reported because the upper platen would often come into contact with the top of the metal fixture before the ultimate failure of a specimen. In addition, it is noteworthy that both the experimental compression stiffness and strength surpass the experimental tensile stiffness and strength, respectively.

## 4. Fiber Flow Modeling for Stiffness and Strength Prediction

### 4.1. Fiber Orientation Kinematics

Over the past few decades there has been considerable work in the area of fiber motion kinetics and the resulting structural performance of the processed composite. The following section pulls together a summary of the high points along with various components that were used in the present study. Consider a fluid flow domain with the velocity profile **v**(**x**) and velocity gradients **L**(**x**), where **x** denotes position. Jeffery’s foundational work in expressing fiber motion for a dilute suspension in such a domain describes the time rate of change in orientation of a single, rigid, inertialess ellipsoid [2],

(5) $$\begin{array}{cc} \dot{p} & =W\cdot p+\xi \left(D\cdot p-D:ppp\right) \end{array}$$

In the above equation, the material derivative of the unit vector **p**, which points along the long axis of the fiber, is used. That is, $\dot{p}=\frac{dp}{\mathrm{dt}}=\frac{\partial p}{\partial t}+v\cdot \nabla p$. In addition, the vorticity tensor is defined as $W=\frac{1}{2}\left(L-L^{T}\right)$, and the rate of deformation tensor is similarly defined as $D=\frac{1}{2}\left(L+L^{T}\right)$. *ξ* is the fiber geometric term, which allows Equation (5) to be used for other axisymmetric shapes besides ellipsoids as long as an equivalent ellipsoidal aspect ratio, $r_{e}$, is properly defined. Expressing the fiber geometric term *ξ* in terms of $r_{e}$ gives (see, e.g., [15])

(6) $$\begin{array}{c} \xi =\frac{\left(r_{e}^{2}-1\right)}{\left(r_{e}^{2}+1\right)} \end{array}$$

Jeffery’s equation as expressed in Equation (5) may be used in dilute fiber solutions, but a fiber interaction model must be used for concentrated solutions. The concentrated regime may be defined as $V_{f}>\left(d/L\right)$, where $V_{f}$ is the fiber volume fraction and *d* and *L* are the fiber diameter and length. For the carbon fibers in the CF-ABS used in the present study d/L= 6.4 μm/278 μm = 2.3%. The CF-ABS used in the present study had $V_{f}=8.11\%$ (this corresponds to a 13% weight fraction) and 8.11% > 2.3%, thus the CF-ABS flow was considered concentrated and therefore a fiber interaction model was used. In addition, the orientation tensor approach popularized by Advani and Tucker [3] was used to describe the orientation state of a population of fibers and save orders of magnitude in computational time. Cast in terms of orientation tensors, there are multiple options for the proper characterization of the fiber interaction kinetics, such as the retarding principal rate model from Tseng et al. [16], the interaction coefficient approach of Folgar and Tucker [4], and the anisotropic rotary diffusion model of Phelps and Tucker [6]. In the present study, we focused on the fiber interaction model of Wang et al. [5],

(7) $$\begin{array}{c} \dot{A}=W\cdot A-A\cdot W+\xi \left\{D\cdot A+A\cdot D-2\left[A_{4}+\left(1-\kappa \right)\left(L_{4}-M_{4}:A_{4}\right)\right]:D\right\}\\ +2\kappa C_{I}\dot{\gamma }\left(I-3A\right)\;\; \end{array}$$

where the second- and fourth-order orientation tensors, as discussed by Advani and Tucker [3], are defined as

(8) $$\begin{array}{c} A=\oint pp\psi \left(p\right)dp,\;\;\;\;\;A_{4}=\oint pppp\psi \left(p\right)dp \end{array}$$

In the above, ψ(p) is the orientation probability density function. In addition, *κ* is the slowness factor as discussed by Wang et al. [5], $C_{I}$ is the fiber interaction constant introduced by Folgar and Tucker [4], $\dot{\gamma }$ is the scalar magnitude of **D**, $L_{4}\;=\;\sum_{i=1}^{3}\lambda_{i}e_{i}e_{i}e_{i}e_{i}$, $M_{4}\;=\;\sum_{i=1}^{3}e_{i}e_{i}e_{i}e_{i}$, and $\lambda_{i}$ and $e_{i}$ are the *i*th eigenvalue and eigenvector of **A**. Based on the work of Bay and Tucker [17], we take

(9) $$\begin{array}{c} C_{I}=1.84\times 10^{-2}\exp\left(-0.7148a_{r}V_{f}\right) \end{array}$$

Note, that $a_{r}$ is the geometric fiber aspect ratio ($a_{r}=L/D$) and is related to the equivalent ellipsoidal aspect ratio of a cylindrical fiber as given by Zhang et al. [15].

(10) $$\begin{array}{c} r_{e}=0.000035a_{r}^{3}-0.00467a_{r}^{2}+0.764a_{r}+0.404 \end{array}$$

Furthermore, based upon earlier work by the present authors (see, e.g., [8]), a value for the slowness parameter of κ=0.125 was selected. Finally, the eigenvalue-based orthotropic closure discussed by Wetzel [18] and VerWeyst [19] was used to estimate $A_{4}$ due to its accuracy and efficiency. Equation (7) and all the equations in this section which it depends on were custom-coded by the authors in MATLAB, such that all flow kinematics were performed in the MATLAB environment. This was conducted along several streamlines in the flow domain to capture a fine-resolution solution for the fiber orientation state across the flow domain.

### 4.2. Fiber Micromechanics

The linear elastic nature of a material is often described using Hooke’s law to relate the stress, $\sigma_{ij}$, to the strain, $\epsilon_{ij}$, through the stiffness, $C_{ijkl}$, or the compliance, $S_{ijkl}$, as

(11) $$\begin{array}{c} \sigma_{ij}=C_{ijkl}\epsilon_{kl},\;\;\;\;\;\;\;\;\;\epsilon_{ij}=S_{ijkl}\sigma_{kl} \end{array}$$

Fibers and matrix have different values for their respective stiffness and due to the length scales considered, an effective stiffness is considered for the composite, $\langle C_{ijkl}\rangle$. This effective stiffness, also termed the homogenized stiffness, due to its relationship to the associated underlying unidirectional stiffness tensor of the composite, ${\bar{C}}_{ijkl}$, and the fiber orientation distribution function, is expressed as

(12) $$\begin{array}{c} \langle C_{ijkl}\rangle =\int_{0}^{2\pi }\int_{0}^{\pi }{\bar{C}}_{ijkl}\psi \left(\theta ,\phi \right)\sin\theta d\theta d\phi \end{array}$$

The underlying unidirectional stiffness tensor of the associated composite ${\bar{C}}_{ijkl}$ may be found in a variety of methods, the Halpin–Tsai and rule of mixtures often being the most popular due to their ease of implementation. In the present study, the authors used the Tandon and Wang [20] model with the closed-form solution suggested in Tucker and Liang [21] and with the mathematical form expressed in Zhang [22]. The choice of the Tandon and Wang model for the underlying unidirectional stiffness tensor of the associated composite was based upon the results from [21] in comparison to other available unidirectional composite models. Thus, with knowledge of the underlying stiffness tensor ${\bar{C}}_{ijkl}$ and the fiber orientation state from Equation (7), the effective stiffness tensor at a point could be computed. Advani and Tucker [3] provided a mathematical form of the effective stiffness tensor in terms of the orientation tensors as

(13) $$\begin{array}{ccc} \langle C_{ijkl}\rangle \;\;\; & = & \;\;\;B_{1}A_{ijkl}+B_{2}\left(A_{ij}\delta_{kl}+A_{kl}\;\delta_{ij}\right)+B_{3}\left(A_{ik}\delta_{jl}+A_{il}\delta_{jk}+A_{jl}\delta_{ik}+A_{jk}\delta_{il}\right)\\ \;\;\; & & \;\;\;+B_{4}\left(\delta_{ij}\delta_{kl}\right)+B_{5}\left(\delta_{ik}\delta_{jl}+\delta_{il}\delta_{jk}\right) \end{array}$$

where the coefficients $B_{i}$ can be cast in terms of the underlying unidirectional stiffness tensor ${\bar{C}}_{ijkl}$ as presented in [3]. Observe that in Equation (13), the fourth-order orientation tensor $A_{ijkl}$ appears. The same orthotropic closure of VerWeyst [19] and Wetzel [18] used in Equation (7) is also used in Equation (13).

The Tsai–Wu [23] model for the onset of failure was used and is expressed in terms of the second-order and fourth-order strength tensors, respectively, $f_{i}$ and $F_{ij}$, as

(14) $$\begin{array}{c} f_{m}\sigma_{m}+F_{mn}\sigma_{m}\sigma_{n}=1 \end{array}$$

In Equation (14), a contracted notation is used where the index pairs [11, 22, 33, 23, 13, 12] are replaced by [1, 2, 3, 4, 5, 6]. For example, the stress tensor in index notation $\sigma_{ij}$ can be expressed in contracted notation as $\sigma_{m}$ where $\left[\sigma_{11},\;\sigma_{22},\;\sigma_{33},\;\sigma_{23},\;\sigma_{13},\;\sigma_{12}\right]\to \left[\sigma_{1},\;\sigma_{2},\;\sigma_{3},\;\sigma_{4},\;\sigma_{5},\;\sigma_{6}\right]$. In Equation (14), the summation convention continues to be used where $\left\{m,\;n\right\}\in \left\{1,\;2,\;\ldots ,\;6\right\}$. Alternatively, the failure envelope in terms of the contracted strain tensor is

(15) $$\begin{array}{c} g_{m}\epsilon_{m}+G_{mn}\epsilon_{m}\epsilon_{n}=1 \end{array}$$

where $g_{m}$ and $G_{mn}$ are, respectively, the contracted second- and fourth-order strength tensors. One can obtain the relationship between the tensors $f_{m}$, $F_{mn}$, $g_{m}$, and $G_{mn}$ as (see e.g., [23])

(16) $$\begin{array}{c} g_{m}=f_{o}C_{om},\;\;\;G_{mn}=F_{op}C_{om}C_{pn} \end{array}$$

where the summations on the indices *o* and *p* are from one to six, and $C_{mn}$ is the contracted form of the stiffness tensor discussed in Equation (13). As discussed in [8], the strength tensor relationship for the failure envelope can be recast in terms of the underlying unidirectional strength tensors, ${\bar{f}}_{m}$, ${\bar{F}}_{mn}$, ${\bar{g}}_{m}$, and ${\bar{G}}_{mn}$, where the bar indicates the unidirectional composite property, using a homogenization similar to that of the stiffness tensor of Equation (12) as

(17) $$\begin{array}{c} {\langle g\rangle }_{m}\epsilon_{m}+{\langle G\rangle }_{mn}\epsilon_{m}\epsilon_{n}=1 \end{array}$$

For a unidirectional-fiber-reinforced composite with all fibers aligned along the $x_{1}$ axis, the unidirectional strength tensors ${\bar{f}}_{m}$ and ${\bar{F}}_{mn}$ may be expressed in terms of the tensile and compression strengths in the form

(18) $$\begin{array}{ccc} {\bar{f}}_{1}\;\; & = & \;\;\frac{1}{{\bar{\sigma }}_{LT}}-\frac{1}{{\bar{\sigma }}_{LC}},\;\;{\bar{f}}_{2}={\bar{f}}_{3}=\frac{1}{{\bar{\sigma }}_{TT}}-\frac{1}{{\bar{\sigma }}_{TC}},\;\;{\bar{F}}_{11}=\frac{1}{{\bar{\sigma }}_{LT}{\bar{\sigma }}_{LC}},\\ {\bar{F}}_{22}\;\; & = & \;\;{\bar{F}}_{33}=\frac{1}{{\bar{\sigma }}_{TT}{\bar{\sigma }}_{TC}},\;\;{\bar{F}}_{44}=2\left({\bar{F}}_{22}-{\bar{F}}_{23}\right),\;\;{\bar{F}}_{55}={\bar{F}}_{66}=\frac{1}{\tau^{2}} \end{array}$$

where the first subscript of the term $\bar{\sigma }$ indicates the longitudinal (*L*) axis along the fiber or the transverse (*T*) axis, and the second subscript indicates a tensile (*T*) load or compression (*C*) load. For example, ${\bar{\sigma }}_{TC}$ is the experimental transverse strength found from compression loading. In the present study, we assumed the tensile and compression behavior for the transverse load direction of the underlying unidirectional composite was identical, ${\bar{\sigma }}_{TT}={\bar{\sigma }}_{TC}=\sigma_{m}$, where $\sigma_{m}$ is the experimentally obtained strength of the matrix. We also assumed the corresponding shear strength of the matrix was found as $\tau =\sigma_{m}/\sqrt{3}$. Using the form suggested in [24], the remaining nonzero terms for ${\bar{F}}_{mn}$ were expressed as ${\bar{F}}_{12}={\bar{F}}_{13}=-{\bar{F}}_{11}/4$ and ${\bar{F}}_{23}=-{\bar{F}}_{22}$. Then, using Equation (16), the unidirectional strength tensors ${\bar{g}}_{m}$ and ${\bar{G}}_{mn}$ were obtained. As shown in [8], the homogenized fourth-order strength tensor was found using an identical form as Equation (13) in terms of the orientation tensors. The homogenized second-order strength tensor in terms of the second-order orientation tensor and second-order strength tensor of the underlying unidirectional SFRP was found as (see, e.g., [25])

(19) $$\begin{array}{ccc} {\langle g\rangle }_{1}\;\;\; & = & \;\;\;\left({\bar{g}}_{1}\;-\;{\bar{g}}_{2}\right)A_{11}\;+\;{\bar{g}}_{2},\;\;{\langle g\rangle }_{2}\;=\;\left({\bar{g}}_{1}\;-\;{\bar{g}}_{2}\right)A_{22}\;+\;{\bar{g}}_{2},\;\;{\langle g\rangle }_{3}\;=\;\left({\bar{g}}_{1}\;-\;{\bar{g}}_{2}\right)A_{33}\;+\;{\bar{g}}_{2}\\ {\langle g\rangle }_{4}\;\;\; & = & \;\;\;\left({\bar{g}}_{1}\;-\;{\bar{g}}_{2}\right)A_{23},\;\;{\langle g\rangle }_{5}\;=\;\left({\bar{g}}_{1}\;-\;{\bar{g}}_{2}\right)A_{13},\;\;{\langle g\rangle }_{6}\;=\;\left({\bar{g}}_{1}\;-\;{\bar{g}}_{2}\right)A_{12} \end{array}$$

In the present study, the composite was considered at the edge of failure when the equality of Equation (17) was satisfied. For simulation purposes, a loading state was imposed on the composite and the following expression was evaluated

(20) $$\begin{array}{c} \varphi \left(\epsilon \right)={\langle g\rangle }_{m}\epsilon_{m}+{\langle G\rangle }_{mn}\epsilon_{m}\epsilon_{n} \end{array}$$

where ***ϵ*** is the strain state from the imposed load on the structure. The SFRP is not predicted to fail as long as $\varphi \left(\epsilon \right)<1$. When $\varphi \left(\epsilon \right)$ reaches one, the SFRP is predicted to fail.

The longitudinal tensile strength ${\bar{\sigma }}_{LT}$ for the unidirectional composite was found using the modified rule of mixtures suggested by Van Hattum and Bernardo [25] as

(21) $$\begin{array}{c} {\bar{\sigma }}_{LT}\left(L/d\right)=V_{f}\frac{\tau L}{d}+\sigma_{m}^{\prime }\left(1-V_{f}\right) \end{array}$$

where the above expression is only valid when the fiber length, *L*, is less than the critical fiber length, $L_{c}$. The fibers in the current study had a typical fiber length of 200–300 μm in the final processed composite structure, whereas the critical fiber length assumed in this study, $L_{c}≃0.9$ mm (taken from [25]), was much greater than the largest fiber length identified in Figure 6. The term $\sigma_{m}^{\prime }$ is the stress of the matrix at the failure strain of the fiber and is experimentally obtained once the strain at failure for the fiber is known.

The longitudinal compression strength was cast in the form suggested by Hayashi and Koyama [26] as expressed by Bajracharya et al. [27]

(22) $$\begin{array}{c} {\bar{\sigma }}_{LC}=\chi E_{f}\epsilon_{m}^{*}V_{f}+(1-V_{f})\sigma_{m}^{*} \end{array}$$

where $E_{f}$ and $E_{m}$ are the moduli of, respectively, the fiber and the matrix, and $\sigma_{m}^{*}=E_{m}\epsilon_{m}^{*}$, where $\epsilon_{m}^{*}$ is the strain at which the matrix yields. The parameter χ is expressed in terms of the weight-average length of the fibers, $L_{w}$, and the critical fiber length, $L_{c}$, where $\chi =L_{w}/\left(2L_{w}\right)$.

It is well known that the presence of porosity in the composite reduces the stiffness and the strength. In the thesis of Nargis [10], the average void fraction identified in her specimen was 13.84%, a value that was used in this paper. Using the estimation of Zhang et al. [28] that the knockdown factor for the stiffness and the tensile strength is the same and is a function of the void volume fraction, the effective stiffness, $E^{eff}$, and strength, $\sigma^{eff}$, were expressed as

(23) $$\begin{array}{c} \frac{E^{eff}}{E_{without\;porosity}}=\frac{\sigma^{eff}}{\sigma_{without\;porosity}}={\left(1-V_{voids}\right)}^{n} \end{array}$$

where in the present study n=2 was used. Equation (23) was used to find the effective stiffness values $E_{LT}^{eff}$, $E_{LC}^{eff}$, and $E_{LF}^{eff}$ and the effective strength values $\sigma_{LT}^{eff}$, $\sigma_{LC}^{eff}$, and $\sigma_{LF}^{eff}$.

### 4.3. Deposition Flow Domain

**Figure 9:**
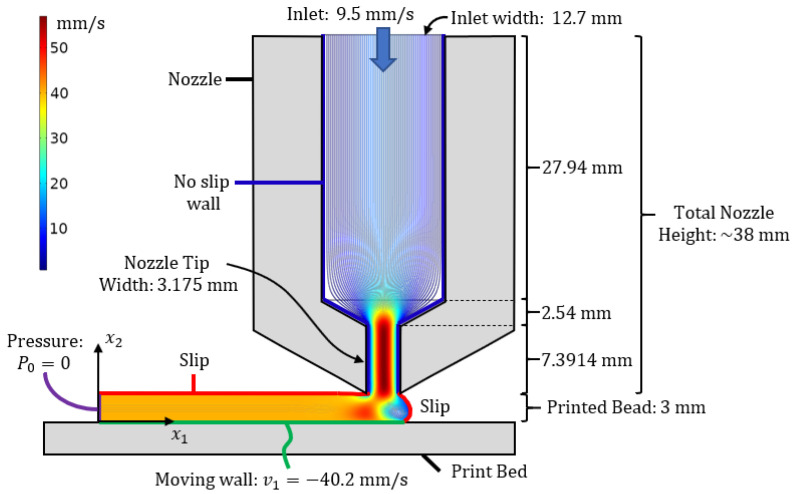
Finite element modeling domain at the tip of the LAAM nozzle including the deposition region.

The flow domain in the present study is depicted in Figure 9 with the dimensions selected based upon the nozzle shown in Figure 2. Solutions for the Navier–Stokes equations were performed using COMSOL Multiphysics. The interior walls of the nozzle were assumed to be no-slip boundary conditions along with the surface of the print bed, whereas the surfaces of the melt flow that are outside of the confined nozzle were considered to have slip boundary conditions. The geometry of the flow domain at the leading edge of the flow was constructed such that the surface was stress-free, and the normal component of the velocity was also zero using the approach from Heller et al. [29] to identify the surface using an iterative approach. Once the velocity field was obtained from the finite element simulation, the spatially varying fiber orientation state, $A_{ij}\left(x\right)$, was obtained using Equation (7) with $C_{I}=1.9\times 10^{-3}$, $\kappa \in \left\{0.05,0.2\right\}$, and *λ* cast as a function of $a_{rw}$ of the 3D-printed bead as given in Table 1. The fiber equations of motion were solved numerically using a custom program created in the MATLAB environment written by the authors. Then, using the material properties for the fiber and the matrix listed in Table 4, Equation (20) was evaluated across the flow domain using a custom script created by the authors that took the spatial location and returned the failure parameter at the specified location.

### 4.4. Structural Simulation

**Figure 10:**
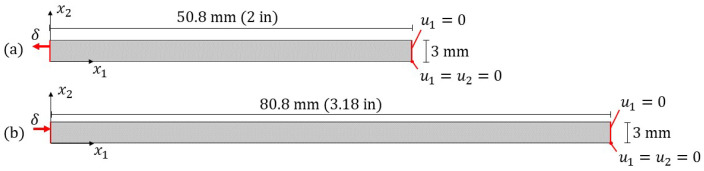
Specimen domains used for the structural analysis for the (**a**) tensile test and (**b**) compression test.

The orientation state at the end of the flow domain, $A_{ij}\left(x_{1}=0,x_{2}\right)$, shown at the origin on the bottom left of Figure 9, was taken as the steady-state orientation and was used for all structural simulations. This orientation state was then projected along the direction of printing to form a specimen with an orientation that changed along $x_{2}$ but was constant along $x_{1}$. The tensile and compression specimen model domains are shown in Figure 10. Each domain is of a single deposited bead. The bead subjected to tension or compression is allowed to freely slide in the vertical direction, except in the lower, right corner. That is, the displacement $u_{2}$ is zero in the lower, right corner and unconstrained everywhere else. The right edge of the bead is fixed horizontally, i.e., $u_{1}=0$, and equal to some static displacement value in the $x_{1}$ direction, *δ*, on the left edge.

**Figure 11:**
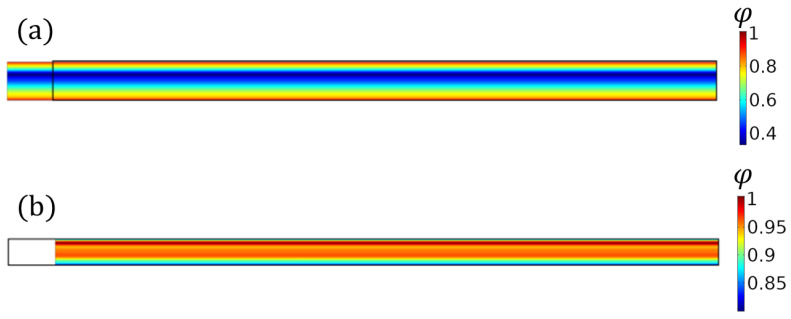
Structural simulation results showing the failure parameter $\varphi \left(x\right)$ for the (**a**) tensile test and (**b**) compression test.

For the applied displacement *δ*, the spatially varying strain, ***ϵ***(**x**), was computed from the finite element results along with the surface stresses. The effective stiffness values for tension or compression, $E_{LT}^{eff}$ and $E_{LC}^{eff}$, respectively, were obtained by taking the average of the stress divided by the applied strain on the right end of the loaded specimen. Then, using the spatially varying strain values along with Equation (20), the spatially varying value for $\varphi \left(\epsilon \right)$ was obtained at every point in the solution domain. If the maximum value for φ was less than one, then the applied displacement *δ* was increased, whereas if φ at any point in the domain was more than one, the applied load was decreased. This process continued until the maximum spatial value for *φ* was one, thus suggesting that the member was at the onset of failure. At this point, $\sigma_{LT}^{eff}$ and $\sigma_{LC}^{eff}$ were calculated. A representative solution is shown in Figure 11a,b for a displacement resulting in a tensile and compression failure, respectively.

The finite element process described above was extended to study the stiffness and strength response over a range of values for *κ*. This first study fixed the fiber length and diameter to be that in the deposited bead, yielding a weight-average fiber aspect ratio of 38.8 as given in Table 1. The fibers were assumed to be randomly orientated initially for the flow domain of Figure 9, and once deposited, the structural domain shown in Figure 10 was analyzed for the stiffness and strength estimates for tension and compression. Using the fiber aspect ratio of 38.8 and a weight fraction of 13% (i.e., a volume fraction of 8.11%), the interaction coefficient from Equation (9) evaluated to $C_{I}=1.9\times 10^{-3}$. The range for κ was taken to be 0.05 to 0.2, within the range suggested by Wang et al. [5]. The remaining modeling inputs in this study are given in Table 4.

**Figure 12:**
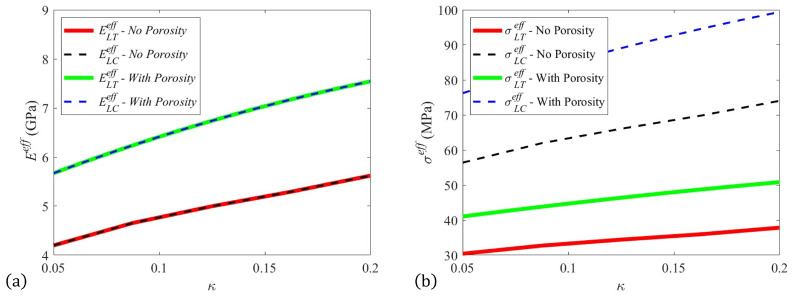
Effective stiffness and strength predictions of the deposited bead for $a_{r}=38.8$. (**a**) Effective stiffness and (**b**) effective strength.

The results for the stiffness and strength parameters as a function of fiber slowness parameter *κ* are provided in Figure 12 for both the scenario when the porosity is neglected and the scenario when the porosity is accounted for in the structural simulations. Notice in Figure 12 that the tensile and compression stiffness values are graphically indistinguishable regardless of the porosity. This is by construction in the model, as the stiffness response is assumed to be equivalent in tension and compression. Conversely, the compression strength is measurably more than that of the tensile strength. Observe that the stiffness and strength increase as a function of the increasing value of the slowness parameter. This correlates to the increase in the orientation. As the slowness parameter, *κ*, increases from zero to one, the rate of alignment increases as well; thus, a higher value of the parameter *κ* correlates with a higher value of the alignment along the flow direction, and thus a higher value for the stiffness and strength for fibers that are stiffer and stronger than the surrounding matrix. It is also worth noting the significant reduction in the part performance as a result of porosity. This highlights the importance of accounting for porosity and the need to identify a means to reduce porosity generation in the deposited bead during processing.

The last modeling study focused on the importance of including the reduction in the fiber length in the structural simulations. Taking the three length distributions presented in Table 1 for the case of the pellet, the extrudate, and the deposited bead, the weight-average length is shown to decrease from 424 μm to 348 μm to 279 μm. Performing the flow simulation with the corresponding fiber aspect ratios, followed by the structural simulations led to the stiffness and strength predictions provided in Table 5. Observe that by properly including the reduction in the fiber length, there is a reduction of nearly 12% in the stiffness, a reduction of over 30% in the tensile strength, and a reduction of over 14% in the compression strength.

## 5. Comparison between Model and Experiment

Table 6 summarizes the experimental results of this study and Table 7 summarizes the model predictions for comparison. These results show that the model predictions are on the high side of the experimental results. However, there are several possible culprits for this.

On the experimental side, the top surfaces of the specimens were machined down to 10–20% of the specimen height to eliminate waviness and to ensure uniform cross sections. While uniform cross sections are desirable, the skin of the specimens contained a high fiber alignment in the load-bearing direction. Thus, removing this skin from the tops of the specimens could have potentially caused a noticeable drop in the mechanical properties.

On the modeling side, Table 7 shows that a relatively large range of predictions can be found depending on the parameters fed into the models. The lower range for $E_{LT}^{eff}$ and $E_{LC}^{eff}$ should be selected in the present study based on the recommendations found in recent fiber interaction model papers to decrease the value of *κ*. *κ* has a greater effect than $C_{I}$, but there are other parameters that were estimated in the present study that could also influence the accuracy of the models. These include the initial orientation state and the constituent material properties in Table 4. Thus, it is important to zero in on accurate modeling parameters when using the proposed models in order to tighten the prediction window. In addition, the recent work by Nargis and Jack in [11] showed that the porosity was found to be significantly larger than anticipated, coupled with a broad spectrum of void sizes. Thus, accounting for porosity and selecting the lower range for the slowness factor *κ*, the predicted value for the compressive stiffness, $E_{LC}^{eff}$, came out to be 4.2 GPa, whereas the measured value was 4.06 GPa; the predicted value for the tensile stiffness, $E_{LT}^{eff}$, was 4.2 GPa, whereas the measured value was 2.71 GPa; the predicted value for the compressive strength $\sigma_{LC}^{eff}$, was 56 MPa, whereas the measured value was 59 MPa; and the predicted value for the tensile strength, $\sigma_{LT}^{eff}$, was 30 MPa, whereas the measured value was 27 MPa. Each of these values, with the exception of the tensile stiffness, is well within an acceptable range. This study highlights a limitation of the present modeling that should be addressed in a future study. The present model assumes that the tensile and compressive stiffness are identical, which is not the case based upon the results presented. Thus, a future expansion of the model to allow for discontinuous tensile and compressive behavior will be required prior to full-scale industrial implementation and will be the scope of a future study.

## 6. Conclusions

Having so many modeling parameters increases the difficulty to fully validate the proposed models. However, the results provided indicate the modeling framework yields results for the effective strength and stiffness that align with experimental characterization to within ∼1% and ∼10% for the longitudinal compressive and tensile strength, respectively, and to within ∼3% and ∼50% for the longitudinal compressive and tensile stiffness, respectively. In addition, a framework has been developed that allows for improvement by model substitution as more accurate modeling inputs are found. The modeling methodology goes from the properties of the individual fibers and surrounding matrix, through the fiber kinematic equations of motion through the final part performance. Results show the sensitivity of the final predicted response to subtle changes in the fiber interaction behavior as well as a reduction in the final part performance through the incorporation of the known porosity of the final processed part.

For future work on the experimental side, improvement in specimen preparation should be sought. For example, nonmachined, multibead tensile and compression specimens could be used to reduce the effect anomalous defects might have, or injection-molded specimens (which have less void content) could be used to reduce the effects of porosity. On the modeling side, in general, efforts to isolate and independently verify each underlying model and model input would be helpful. The fiber orientation could be physically measured as in Nargis’ work [10], for example, and directly input into the stiffness and strength models to validate these models independently from a fiber orientation model. In addition, it is well understood that polymers are sensitive to changes in temperature, and performing tests across a temperature range would be of interest. The present model is linearly elastic, and extending the work to viscoelastic models would be of interest. A first pass at a 3D model for stiffness prediction which incorporated Nargis’ results was performed in [12], but this area still needs more work. In addition, the effects of porosity need to be accounted for better, both in modeling the fiber orientation state and predicting the final mechanical properties as a function of the orientation state. The flow and orientation kinematics relationship was decoupled in the present study, but coupling them may make a difference in the predicted final orientation state. Another consideration for a future study may be a fiber aspect ratio distribution, rather than a single-value aspect ratio.

## Figures and Tables

**Table 1:** Fiber length and aspect ratio data, corrected for fiber degradation.

Parameter	Unit	Pellet	Extrudate	3D-Printed Bead
$L_{n}$	μm	293	271	169
$L_{w}$	μm	424	348	279
$d_{n}$	μm	7.83	7.51	7.19
$d_{w}$	μm	7.83	7.51	7.20
$a_{rn}$	μm	37.4	36.1	23.5
$a_{rw}$	μm	54.1	46.3	38.8

**Table 2:** Tensile testing statistics for 13% CF-ABS specimens.

Property	Unit	Minimum	Maximum	$\bar{x}$	$s_{n-1}$	$C_{V}$
$E_{LT}^{eff}$	GPa	2.56	2.88	2.71	0.163	6.04%
$\sigma_{LT}^{eff}$	MPa	25.9	28.2	27.3	1.04	3.79%
$\sigma_{LT}^{ult}$	MPa	30.5	32.8	32.0	1.04	3.24%

**Table 3:** Compression testing statistics for 13% CF-ABS.

Property	Unit	Minimum	Maximum	$\bar{x}$	$s_{n-1}$	$C_{V}$
$E_{LC}^{eff}$	GPa	3.65	4.42	4.06	0.336	8.28%
$\sigma_{LC}^{eff}$	MPa	45.4	64.7	58.6	7.72	13.2%

**Table 4:** Properties of ABS matrix and carbon fiber used for modeling.

Property	Unit	ABS Matrix	Ref.	Carbon Fiber	Ref.
Elastic modulus	GPa	$E_{m}\;=\;2.41$	[30]	$E_{f}\;=\;218$	[25]
Poisson’s ratio	-	$\nu_{m}\;=\;0.35$	[31]	$\nu_{f}\;=\;0.26$	[25]
Density	kg/m^3^	$\rho_{m}\;=\;1040$	[30]	$\rho_{f}\;=\;1760$	[32]
Dynamic viscosity	Pa·s	$\mu_{m}\;=\;3200$	[33]	-	-
Tensile yield strength	MPa	$\sigma_{m}\;=\;37.9$	[30]	$\sigma_{f}\;=\;6024$	[25] ^†^
Compression yield strain	$\frac{m}{m}$	$\epsilon_{m}^{*}\;=\;0.04$	[34]	-	-
Matrix stress at fiber failure strain	MPa	$\sigma_{m}^{\prime }\;=\;24$	[32,35] ^‡^	-	-
Shear strength	MPa	τ=21.9	[25] ^†^	-	-
Fiber length	μm	-	-	$L_{f}\;=\;279$	^ †† ^
Fiber diameter	μm	-	-	$d_{f}\;=\;7.20$	^ †† ^
Geometric aspect ratio	$\frac{m}{m}$	-	-	$a_{r}\;=\;38.8$	^ †† ^
Ellipsoidal aspect ratio	$\frac{m}{m}$	-	-	$r_{e}\;=\;25.0$	[15] ^†^
Critical fiber length	μm	-	-	$L_{c}\;=\;835$	[25] ^†^
Fiber weight fraction	$\frac{\mathrm{kg}}{\mathrm{kg}}$	-	-	$w_{f}\;=\;0.13$	[36]
Fiber volume fraction	$\frac{m^{3}}{m^{3}}$	-	-	$V_{f}\;=\;8.11\%$	[37] ^†^

^†^ These references are for the equations used to calculate the property. ^‡^ Fiber failure strain taken from [32] and using the stress–strain curve from [35] for ABS, the matrix stress was obtained. ^††^ Refers to experimental results in the present paper.

**Table 5:** Tensile and compression stiffness and strength predictions as a function of the weight-average aspect ratios from Table 1 along the flow domain for κ=0.125.

Parameter	$E_{\mathrm{LT}}^{\mathrm{eff}}=E_{\mathrm{LC}}^{\mathrm{eff}}$ (GPa)	$\sigma_{\mathrm{LT}}^{\mathrm{eff}}$ (MPa)	$\sigma_{\mathrm{LC}}^{\mathrm{eff}}$ (MPa)
$L_{w}\;=\;424\;$μm (pellet)	5.58	44.8	75.4
$L_{w}\;=\;348\;$μm (extrudate)	5.34	39.3	71.2
$L_{w}\;=\;279\;$μm (bead)	5.00	34.4	66.1

**Table 6:** Summary of experimental results, with the error taken from the standard deviation of experimental results.

Property	Average Property
$E_{LT}^{eff}$	2.71 ± 0.16 GPa
$E_{LC}^{eff}$	4.06 ± 0.34 GPa
$\sigma_{LT}^{eff}$	27.3 ± 1.0 MPa
$\sigma_{LC}^{eff}$	58.6 ± 7.7 MPa

**Table 7:** Tensile and compression stiffness and strength predictions of the deposited bead using the RSC model with a random initial orientation state ($A_{ij}=\frac{1}{3}\delta_{ij}$), $a_{rw}=38.8$, and $\kappa \in \left\{0.05,0.20\right\}$. Equation (23) was used to generate the second row from the first row.

Accounting for Porosity?	$E_{\mathrm{LT}}^{\mathrm{eff}}=E_{\mathrm{LC}}^{\mathrm{eff}}$ (GPa)	$\sigma_{\mathrm{LT}}^{\mathrm{eff}}$ (MPa)	$\sigma_{\mathrm{LC}}^{\mathrm{eff}}$ (MPa)
No	5.67–7.54	41.0–50.8	76.2–99.4
Yes	4.20–5.62	30.4–37.8	56.4–74.0

## Abbreviations

The following abbreviations are used in this manuscript:

ABS	Acrylonitrile butadiene styrene
ASTM	American Society for Testing and Materials
CF-ABS	Carbon-fiber-reinforced acrylonitrile butadiene styrene
CNC	Computer numerical control
HMI	Human–machine interface
LAAM	Large area additive manufacturing
PDF	Probability distribution function
RPM	Revolutions per min
SFRP	Short-fiber-reinforced polymer
TGA	Thermogravimetric analyzer

## References

[1] Love L., Kunc V., Rios O., Duty C., Elliott A., Post B., Smith R., Blue C. (2014). The Importance of Carbon Fiber to Polymer Additive Manufacturing. J. Mater. Res..

[2] Jeffery G. (1923). The Motion of Ellipsoidal Particles Immersed in a Viscous Fluid. Proc. R. Soc. Lond. A.

[3] Advani S., Tucker C. (1987). The Use of Tensors to Describe and Predict Fiber Orientation in Short Fiber Composites. J. Rheol..

[4] Folgar F., Tucker C. (1984). Orientation Behavior of Fibers in Concentrated Suspensions. J. Reinf. Plast. Compos..

[5] Wang J., O’Gara J., Tucker C. (2008). An Objective Model for Slow Orientation Kinetics in Concentrated Fiber Suspensions: Theory and Rheological Evidence. J. Rheol..

[6] Phelps J., Tucker C. (2009). An Anisotropic Rotary Diffusion Model for Fiber Orienation in Short- and Long Fiber Thermoplastics. J. Non-Newton. Fluid Mech..

[7] Sommer D., Favaloro A., Pipes R. (2018). Coupling anisotropic viscosity and fiber orientation in applications to squeeze flow. J. Rheol..

[8] Russell T., Jack D. (2021). Strength prediction in single beads of large area additive manufactured short-fiber polymers. Polym. Compos..

[9] Heller B. (2019). Non-Isothermal Non-Newtonian Flow and Interlayer Adhesion in Large Area Additive Manufacturing Polymer Composite Deposition. Ph.D. Thesis.

[10] Nargis R. (2021). Internal Fiber Orientation Measurements and Void Distribution for Large Area Additive Manufactured Parts Using Optical and SEM Imaging Techniques. Master’s Thesis.

[11] Nargis R., Jack D. (2023). Fiber Orientation Quantification for Large Area Additively Manufactured Parts Using SEM Imaging. Polymers.

[12] Russell T. (2021). Mechanical and Thermal Property Prediction in Single Beads of Large Area Additive Manufactured Short-Fiber Polymer Composites. Ph.D. Thesis.

[13] (2022). Standard Test Method for Tensile Properties of Plastics.

[14] (2015). Standard Test Method for Compressive Properties of Rigid Plastics.

[15] Zhang D., Smith D., Jack D., Montgomery-Smith S. (2011). Numerical Evaluation of Single Fiber Motion for Short-Fiber-Reinforced Composite Materials Processing. J. Manuf. Sci. Eng..

[16] Tseng H.-C., Chang R.-Y., Hus C.-H. (2013). Phenomenological Improvements to Predictive Models of Fiber Orientation in Concentrated Suspensions. J. Rheol..

[17] Bay R., Tucker C. (1992). Stereological Measurement and Error Estimates for Three-Dimensional Fiber Orientation. Polym. Eng. Sci..

[18] Wetzel E. (1999). Modeling Flow-Induced Microstructure of Inhomogeneous Liquid-Liquid Mixtures. Ph.D. Thesis.

[19] VerWeyst B. (1998). Numerical Predictions of Flow Induced Fiber Orientation in Three-Dimensional Geometries. Ph.D. Thesis.

[20] Tandon G., Weng G. (1984). The Effect of Aspect Ratio of Inclusions on the Elastic Properties of Unidirectionally Aligned Composites. Polym. Compos..

[21] Tucker C., Liang E. (1999). Stiffness Predictions for Unidirectional Short-Fiber Composites: Review and Evaluation. Compos. Sci. Technol..

[22] Zhang C. (2011). Modeling of Flexible Fiber Motion and Prediction of Material Properties. Master’s Thesis.

[23] Tsai S., Wu E. (1971). A General Theory of Strength for Anisotropic Materials. J. Compos. Mater..

[24] DeTeresa S., Larsen G. (2001). Derived Interaction Parameters for the Tsai-Wu Tensor Polynomial Theory of Strength for Composite Materials.

[25] Van Hattum J., Bernardo C. (1999). A Model to Predict the Strength of Short Fiber Composites. Polym. Compos..

[26] Hayashi T., Koyama K. (1972). Theory and Experiments of Compressive Strength of Unidirectionally Fiber-reinforced Composite Materials. Mech. Behav. Mater..

[27] Bajracharya R., Manalo A., Karunasena W., Lau K. (2016). Experimental and Theoretical Studies on the Properties of Injection Moulded Glass Fibre Reinforced Mixed Plastics Composites. Compos. Part A.

[28] Zhang Y., Rodrigue D., Ait-Kadi A. (2003). High Density Polyethylene Foams. III. Tensile Properties. J. Appl. Polym. Sci..

[29] Heller B., Smith D., Jack D. (2019). Planar Deposition Flow Modeling of Fiber Filled Composites in Large Area Additive Manufacturing. Addit. Manuf..

[30] Polyhedron Laboratories Inc. (2021). ABS Plastics Testing Services—ABS Testing—ABS Plastic Strength Test.

[31] Engineers Edge LLC (2021). Specifications for Common Plastic Molding Design Material.

[32] Minus M., Kumar S. (2005). The Processing, Properties, and Structure of Carbon Fibers. JOM J. Miner. Met. Mater. Soc..

[33] Russell T., Heller B., Jack D., Smith D. (2018). Prediction of the Fiber Orientation State and the Resulting Structural and Thermal Properties of Fiber Reinforced Additive Manufactured Composites Fabricated Using the Big Area Additive Manufacturing Process. J. Compos. Sci..

[34] Wang J., Xu D., Sun W., Du S., Guo J., Xu G. (2019). Effects of Nozzlebed Distance on the Surface Quality and Mechanical Properties of Fused Filament Fabrication Parts. IOP Conf. Ser. Mater. Sci. Eng..

[35] Banjanin B., Vladic G., Pal M., Balos S., Dramicanin M., Rackov M., Knezevic I. (2018). Consistency Analysis of Mechanical Properties of Elements Produced by FDM Additive Manufacturing Technology. Materia.

[36] MatWeb LLC (2021). Avient Stat-Tech AS-13CF/000 Black Acrylonitrile Butadiene Styrene (ABS).

[37] Barbero E. (2017). Introduction to Composite Material Design.

